# Breast Implant Capsule: Friend, Not Foe

**DOI:** 10.1093/asjof/ojac006

**Published:** 2022-01-29

**Authors:** Gianfranco Frojo, William P Adams

**Affiliations:** Department of Plastic Surgery, UT Southwestern Medical Center, Dallas, TX, USA

We read with interest the Letter to the Editor entitled, “Breast Implant Capsule: Are You Going to Leave It In?,” ^1^ originally written in response to the article “Subpectoral Implant Repositioning With Partial Capsule Preservation: Treating the Long-Term Complications of Subglandular Breast Augmentation.” ^2^ The authors present the case of a patient with a history of revisionary breast surgery with equivocal breast imaging prompting further surgery to rule out malignant pathology in the bilateral upper breast quadrants that were identified as benign residual capsules with associated calcified hematoma. The authors summarize the clinical conundrum presented by the residual breast capsules and highlight the lack of high-level evidence to guide clinical decision making regarding breast implant capsulectomy. The authors conclude with a recommendation to perform total precise capsulectomy “in cases of implant removal and pocket conversion when the capsule has no role in supporting the new implants.” The authors further purport that the lack of evidence regarding the natural history of residual periprosthetic breast capsules over time warrants capsulectomy as “not all the capsules left in the breast undergo resorption and indeed can lead to diagnostic challenges and unnecessary secondary surgeries surgical.”

We submit a different perspective on this subject and recommend to perform an individualized assessment of each patient’s indications for total precise capsulectomy with thorough understanding by not only the surgeon but also the patient. The decision to perform a capsulectomy is a complex process that should consider the position of the implant pocket, reason for explantation, history of a textured device, potential for capsular pathology, capsular imaging with ultrasound and/or MRI, patient preference, physical examination, and morbidity risk assessment.^3^ It should be noted that a new capsule frequently forms from clinically undetectable seromas postoperatively after performing a complete capsulectomy. Any definitive recommendation regarding capsulectomy in a healthy patient with history and examination findings that do not suggest capsular pathology should be weighed against the risks and potential morbidity related to performing a total precise capsulectomy.^4^ Even in the patients with a history of a previous textured implant device, the potential benefit of a total precise capsulectomy in the setting of a benign capsule has yet to be clearly delineated.^5^ Despite the lack of a high level of evidence to guide clinical decision making related to capsulectomy, anecdotal case reports of patients with complex clinical presentations related to residual capsules do not validate the performance of capsulectomy without careful consideration of all factors and discussion with the patient. Indiscriminate performance of total precise capsulectomy on every patient may potentially lead to unnecessary morbidity including devastating complications such as pneumothorax or less severe untoward outcomes such as aesthetic deformity.^6^ On the other hand, there are advantages to capsule preservation including obviating further surgical risk from a more invasive procedure, preservation of vascularity without disruption of the overlying soft tissue envelope, decreased anesthetic exposure, and adding the potential for other surgical options such as simultaneous implant exchange with fat grafting (SEIF).^4,7^ In the rare clinical scenario that the decision to perform capsulectomy or not is not evident preoperatively, the surgeon may elect to perform a partial or total precise capsulectomy intraoperatively should there be any signs of abnormal capsular appearance at the time of implant removal or exchange. We acknowledge the complexity of determining the need for capsulectomy when the preoperative data are unclear, but each patient and clinical situation should be approached with caution and decisions individualized.
